# Gender inclusive sporting environments: the proportion of women in non-player roles over recent years

**DOI:** 10.1186/s13102-021-00290-4

**Published:** 2021-05-28

**Authors:** R Eime, M Charity, B. C Foley, J Fowlie, L. J Reece

**Affiliations:** 1 School of Science, Psychology and Sport, Federation University, Ballarat, Australia; 2 Institute for Health and Sport, Victoria University, Footscray, Australia; 3 Charles Perkins Centre, School of Public Health, Faculty of Medicine and Health, SPRINTER, Prevention Research Collaboration, The University of Sydney, Sydney, Australia

**Keywords:** Community sport, Volunteer, Gender, Coaches, Administrators

## Abstract

**Background:**

Throughout the ecosystem of sport, women have been and continue to be underrepresented at all levels compared to men. The capacity of community-level sport is heavily reliant on the many non-player roles including governance, as well as administration, coaching and officiating. Recently there has been increased attention to improving the gender balance in sport. The aim of this study is to investigate the proportions of women engaged in non-playing roles in sport (2016–2018).

**Methods:**

This study involved secondary analysis of the AusPlay survey, a national population survey, funded by Sport Australia. This study utilised data from people aged 15-years or older about their involvement in non-playing roles in sport, and their demographic data. Survey respondents were asked “During the last 12 months, have you been involved with any sports in a nonplaying role, such as official, coach, referee, administrator, etc?” Analysis of non-player role responses focussed specifically on the top four non-player role categories; coach, official, administrator and manager. Frequency analysis concentrated on the distribution of men and women involvement in a non-player capacity for the three years, with detailed analysis of the most recent year (2018).

**Results:**

In this study of 61,578 Australians there was a higher proportion of men in non-player roles in sport compared to women, across each of the three years (2018: men 55 %, women 46 %). Involvement of women in coaching increased significantly from 38 % to 2016 to 44 % in 2018 (*p* < 0.001). The proportion of women involved in administration roles significantly decreased from a peak of 51 % in 2017 to 46 % in 2018 (*p* < 0.001).

**Conclusions:**

Aligned with strategic policy and investment strategies, there are gradual increased representation of women in non-playing sport, coaching roles. Women are still underrepresented in terms of coaches, officials and administrators, but are more likely to be managers. It is recommended that there is continued mentoring, identification and emphasising of female role models, and further strategies to increase female presence in non-playing roles. We recommend that future research, in line with appropriate gender and cultural-change theories, investigates and discusses the progress of gender equality throughout playing and non-playing role in sport.

## Background

Inclusion throughout all layers of the sport ecosystem, where people have the opportunity to participate to their desired capacity without discrimination, is an important part of a fair society. Participation in sport in both playing (athlete/participant) and non-playing (coaches/officials/ administration/governance) roles are associated with positive individual, social, strategic and economic benefits [1, 2]. However, women and girls have historically been, and continues to be, an underrepresented throughout sport, [3, 4] in both playing and non-playing roles such as coaches and board members [5].

In terms of participation in community sport, males participate at twice the rate of females [3]. In general, it is argued that gender is a highly visible position of inequality in sport [6], and historically this disparity in participation across gender reflects the societal perceptions that sport participation is part of a typical male domain [7]. Further the negative stereotypes about women playing sport affects the sports activities that women and girls participate in and specifically for those sports that at traditionally male dominated or considered suited to males [7]. These negative stereotypes are also amplified with a lack of female representation in sport media, and specifically in traditionally male dominated sports [8].

An international study of gender diversity in sport governance reports that across 45 countries women remain underrepresented as board directors (global average 20 %), board chairs (11 %) and chief executives (16 %). Few countries achieved a critical mass of women in leadership roles [9]. There is strong evidence that gender diversity on corporate boards has a positive impact through a range of processes and business outcomes as well as being important role models for other women [10].

In Australia, grass-roots sports are often community run, not for profit organisations. Across grass-roots sports there are over 2.3 million people [11] involved in non-playing roles, paid or unpaid roles, who collectively contribute to the capacity of the organisation and deliver their sport [12, 13]. The majority of research to date which has focused on the sport workforce refers to them as ‘volunteers’, however, this insinuates that they are not paid for their time, when in fact roles such as coaches, officials and administrators may be paid. As such, in this study will use the collective term ‘non-playing’ roles for both paid and voluntary roles which enable players to participate throughout the sport ecosystem including sport governance, sport administration, coaching and officiating as well as playing.

Within community grass-roots sport, the motivations for involvement in non-playing roles include networking, having a child playing the sport, as well as other extrinsic benefits such as awards and recognition from others [14–17]. Other key drivers to their involvement include a general interest in the sport, and a desire to help others or to give back to a club or community [14, 15]. In addition to the benefits of people in non-playing roles to run sports clubs, their participation can positively impact individuals. For example their involvement can provide a sense of belonging, sense of satisfaction, work-related experience, new relationships and an increased sense of self [14, 18, 19].

Involvement in non-playing roles in sport has historically been dominated by men (aged 35–54 years) with few women involved [11]. The masculine hegemony in sport can influence how gender operates as an organising principle in leadership in sports organisations [5]. As a result women are often overlooked for sports coaching, officiating and governance roles [20–23]. This is sometimes due to a presumption that women sports coaches do not have the same skillset and attributes as male coaches such as toughness, strength, competitiveness, aggressiveness and loudness, and opinions that women are incapable of coaching sport [21, 24]. Even within women’s sport, men are often the head coaches [20]. A lack of role models for women in non-playing sports roles can hinder their involvement [25].

Recently, the importance of gender diversity in non-playing roles in sport from chief executive officers, board members through to coaches and officials has been highlighted by government and as such various policies and strategies have been developed and implemented [26–28]. For example, in Victoria, Australia there are numerous programs aimed at sports organisations tackling gender inequality on and off the playing field. This includes a Five Year Game Plan, which aims to encourage the sports sector to challenge gender stereotypes and encourage more women and girls to reach their full potential, and includes a range of initiatives including funding for female friendly sports infrastructure and quotas for women on boards [28]. Another example of commitment to gender equality in sport policy is in New Zealand, which focuses on.

increasing female participation at all levels, in sport and recreation in addition to recognising the power that gender balance has to positively change social, economic and culture future of countries [29].

Given these recent government initiatives which focus on gender inclusive sports environments, the aim of this study is to investigate the proportions of women engaged in non-playing roles in sport over recent years.

## Methods

This study involved secondary analysis of the AusPlay survey, a national population survey, funded by Sport Australia [30]. It has been conducted annually from 2015 by computer assisted telephone interviewing to find out about participation in sport and other physical activities in Australia. The survey invites adults (15 + years old) to provide participation behaviours, motivations, barriers, use of technology, involvement in non-playing roles and demographic data.

Randomly selected Australians aged 15 and over were interviewed, with additional questions for children being asked of the main respondent when they were parents or guardians of at least one child in their household. The adult (15 plus) with the most recent birthday was selected for interview when a landline telephone was called. For mobile phone contact, the owner of the mobile was selected. The Sample Pages database was used to produce sampling frames for the random generation of phone numbers to call. Weights for the adult (15 plus) data were determined by geographic strata, age, gender and corresponding Estimated Residential Population (ERP) [30].

Survey respondents were asked “During the last 12 months, have you been involved with any sports in a nonplaying role, such as official, coach, referee, administrator, etc?” Each they responded “Yes”, subsequent questions asked them to describe up to three sports they were involved with, and the non-player role or capacity they fulfilled in each sport. Analysis of non-player role responses focussed specifically on popular non-player role categories; namely coach, official, administrator and manager. The definitions of each role are: ‘coach’ refers to coaches, instructors, trainers or teachers; ‘official’ includes roles such as referees, umpires, scorers, timekeeper and line judge; ‘administrator’ consists of committee members and sport administrators; ‘manager’ comprises of team managers and coordinators. If an individual performed in more than one role, these were counted separately.

Frequency analysis concentrated on the distribution of men and women involvement in a non-player capacity for the three years 2016, 2017 and 2018, with detailed analysis of the most recent year (2018). In line with international and national recommendations, 50 % was considered acceptable representation of women and men in sport [27, 31]. Additional testing examined if having children under 15 years of age, being physically active yourself (at least once in past 12 months), or living in different States/Territories of Australia changed this distribution. Being physically active was defined from the question: In the last 12 months did you participate in any physical activities for sport, for exercise, or for recreation.

All analyses were weighted as per AusPlay method instructions and run in STATA 13.

## Results

Table 1 summarises the demographics of the survey participants who indicated that they participated in a non-player role within sport (Table 1). The numbers presented are weighted numbers. There was a total of 61,578 study participants and 8,016 who reported that they were involved in at least one non-player sport roles. Nearly all the non-player role people also had participated in physical activity at least once in the previous year, and this increased from 95.8 to 2016 to 97.1 in 2018. There were more males than females across each of the three years, however the proportion of females increased, whilst the proportion of males decreased from 2016 to 2018. Under half of those in non-player roles had children aged under 15 years. Due to the large, weighted numbers, most significance testing has resulted in significant differences.

**Table 1:** Demographics for those undertaking any non-player role

	2016		2017		2018	
	**n**	**%**	**n**	**%**	**n**	**%**
	**2,849,402**	**100.0**	**2,898,326**	**100.0**	**3,109,126**	**100.0**
**Participate in physical activity**						
** Yes**	**2,730,672**	**95.8**	**2,780,003**	**95.9**	**3,019,215**	**97.1**
** No**	**118,730**	**4.2**	**118,323**	**4.1**	**89,911**	**2.9**
**Gender**						
** Male**	**1,639,394**	**57.5**	**1,631,732**	**56.3**	**1,693,922**	**54.5**
** Female**	**1,210,008**	**42.5**	**1,266,594**	**43.7**	**1,415,204**	**45.5**
**Child(ren) under 15**						
** yes**	**1,172,783**	**41.2**	**1,123,172**	**38.8**	**1,249,709**	**40.2**

**Fig. 1:**
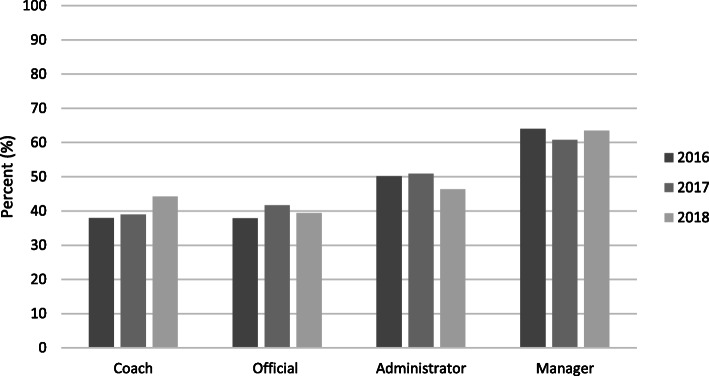
Australian females fulfilling non-player roles in sport: by year

Figure 1 shows the proportion of women involved in sport in non-playing roles across 2016, 2017 and 2018. Involvement of women in coaching increased significantly from 38 % to 2016 to 39 % in 2017 and 44 % in 2018 (*p* < 0.001). Additionally, proportion of women involved in administration roles significantly decreased from 51 % to 2017 to 46 % in 2018 (*p* < 0.001).

**Fig. 2:**
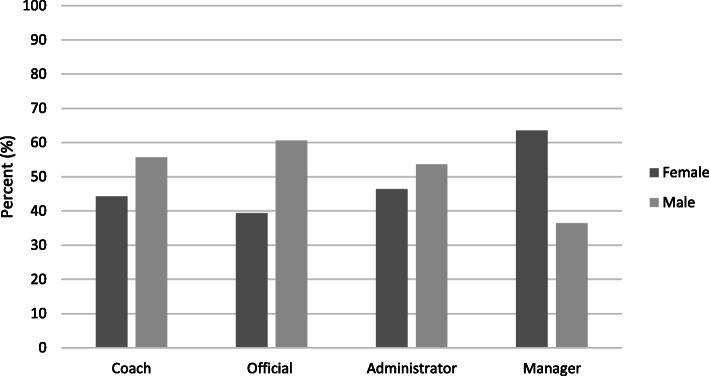
Non-playing roles, 2018: by gender

Further analysis of non-playing roles by gender in 2018 found that managers are most likely to be female (64 %), whereas coaches, officials and administrators are most likely males (56 %, 61 and 54 % respectively) (*p* < 0.001) (Fig. 2).

Table 2 shows the percentage breakdown of men and women in the roles of administrator, coach, official and manager by Australian States/Territories. Most States and Territories in Australia display gender equitable representation in Administration, coaching and officials’ roles in sport. Women were underrepresented as Administrators in Western Australia (38.5 %), Coaches in Victoria (37.9 %), Official’s in Queensland (30.8 %) and Managers in the Northern Territory (27.7 %). Managers were predominantly women in six jurisdictions, with less than one in five managers in Western Australia and Tasmania being men.

**Table 2:** Non-playing roles, 2018: by state and gender

		female		male		person	
**Role**	**State**	**n**	**%**	**n**	**%**	**n**	**%**
Administrator	Australian Capital Territory	4,774	41.7	6,681	58.3	11,455	100.0
	New South Wales	78,674	47.3	87,598	52.7	166,272	100.0
	Northern Territory	2,267	70.4	954	29.6	3,221	100.0
	Queensland	73,713	54.9	60,671	45.1	134,384	100.0
	South Australia	29,993	49.9	30,108	50.1	60,101	100.0
	Tasmania	5,026	43.2	6,618	56.8	11,644	100.0
	Victoria	78,248	41.5	110,413	58.5	188,660	100.0
	Western Australia	29,063	38.5	46,345	61.5	75,407	100.0
Coach	Australian Capital Territory	13,738	52.5	12,424	47.5	26,162	100.0
	New South Wales	227,406	41.8	317,064	58.2	544,470	100.0
	Northern Territory	20,034	85.7	3,349	14.3	23,383	100.0
	Queensland	145,183	46.8	164,978	53.2	310,160	100.0
	South Australia	49,665	46.5	57,185	53.5	106,851	100.0
	Tasmania	21,382	58.1	15,399	41.9	36,781	100.0
	Victoria	147,070	37.9	241,015	62.1	388,085	100.0
	Western Australia	100,970	49.9	101,467	50.1	202,437	100.0
Official	Australian Capital Territory	8,100	40.0	12,169	60.0	20,270	100.0
	New South Wales	140,531	41.1	201,360	58.9	341,891	100.0
	Northern Territory	1,971	63.0	1,158	37.0	3,129	100.0
	Queensland	65,307	30.8	146,394	69.2	211,701	100.0
	South Australia	39,992	48.5	42,524	51.5	82,516	100.0
	Tasmania	9,709	35.9	17,355	64.1	27,064	100.0
	Victoria	118,142	40.6	173,169	59.4	291,312	100.0
	Western Australia	51,629	40.7	75,210	59.3	126,839	100.0
Manager	Australian Capital Territory	8,977	69.9	3,861	30.1	12,838	100.0
	New South Wales	85,881	66.1	43,969	33.9	129,851	100.0
	Northern Territory	867	27.7	2,258	72.3	3,125	100.0
	Queensland	47,053	56.8	35,723	43.2	82,775	100.0
	South Australia	27,226	63.9	15,403	36.1	42,629	100.0
	Tasmania	2,552	84.1	483	15.9	3,035	100.0
	Victoria	79,823	58.5	56,563	41.5	136,386	100.0
	Western Australia	40,113	80.7	9,595	19.3	49,708	100.0

There are different patterns of gender diversity in non-player roles according to having children aged under 15 years. Among people who had children aged 15 years or younger, 72 % of team manager or coordinator roles were fulfilled by women. Whereas 40 % of coaching or officiating roles were fulfilled by women with children, compared to 60 % of men with children. Administrative or committee member roles, were fulfilled by 59 % women and 41 % men, with children.

In all non-playing roles, women had higher participation when they were also a player in the sport. Whereas for men, their participation in non-player roles was higher when they were not a player in the sport.

## Discussion

Historically, men have dominated sport at all levels including as board directors, chief executive officers, officials, coaches as well as players. The aim of this study was to investigate the proportions of women engaged in non-playing roles in sport over recent years. Given the recent policy and strategic developments aimed at achieving gender equity in sport [27, 28], this study provides further insights into the roles available in sport beyond playing, with a focus on gender. Individuals in non-playing roles are critical in building sustainable sporting communities [32]. Additionally, having diversity among roles can improve sport through acceptance and changing traditional values and practises [9].

This current study demonstrates that there has been an increase in involvement of women in coaching over a short period from 2016 to 2018 with 38 % of coaches being women in 2016 compared to 44 % in 2018. The earlier, 2016 figure is similar to that reported in an American study of intercollegiate sports teams, with 35 % of coaches being women [33]. Whilst the proportion of women coaches increasing in Australia is a positive, it is also positive that other cultural and societal acceptance of female coaches seems to be improving. A recent study of male college athletes reported that the gender of their coach was not important, as long as they were a competent coach [22]. However, there are a range of barriers for women in coaching roles including a lack of support, inadequate salary, job insecurity, as well as difficulties in working with parents/spectators and coaching at weekends and evenings [34]. There is growing body of evidence that females in sport benefit from other female role models, both in participation and in coaching or non-player roles, and that female players often prefer female coaches [8, 35].

This current study shows that women’s involvement in administrative roles has decreased from 50 % to 2016 compared to 46 % in 2018, although this shift may not be cause for concern. These roles include committee members as well as sports administrators. The increased proportion of men in administrative roles may suggest a change in historical societal norms. It is not clear why there would be a change in the proportion of women in administrative roles in sport, however it may be that there has been a redistribution of women across the sports, moving from administration to other non-player roles. A study of sport administrators at a higher level national governing bodies, investigated female development in sport administration, and found that interpersonal relationships with supervisors, mentors and access to professional development helped them to progress [36]. Similar strategies which foster female development in administrative and other non-players roles should be implemented to enhance gender equity throughout the sport sector.

Many people involved in non-playing roles, particularly in youth sport, are parents [18]. This study demonstrated that males and females with children (aged under 15) take on different roles. For those with children, females were much more likely to be team managers or coordinates and administrators or committee members and much less likely to be coaches or officials, compared to males. This may be reflected by the societal gender expectations and norms where females are generally the primary care-giver [37]. It may be that coaching roles are more time pertinent especially with structured times for training and competition, whereas other non-playing roles are less structured time dependent, and more flexible and preferred by females.

The results of this study also highlight that the proportion of women in non-player roles in sport do differ across the States and Territories of Australia. This could be related to different policies and strategic priorities across the different jurisdictions. Future research should investigate specific reasons for these state-based differences and what facilitates gender equity in the national sport sector.

Participation in sport in the capacity as a player can also have an indirect influence on the transition of players to coaches and officials for females. We found that for females, their participation in non-playing roles was higher when they were active themselves. It may be that females are more confident to be involved in non-playing roles if they themselves are active too.

Culture in sport has traditionally been masculine and this does influence the number of women in both playing and non-playing roles in sport [5]. The results of this study demonstrate that with an international and Australian specific strategic focus on more women in leadership or non-playing roles in sport, we can see gradual changes, but cultural change does take time. Having said that, because men still hold most of the senior management positions in sport, they therefore still possess most of the power [38]. Recent research suggests that a culture that promotes inclusion of women in sport at all levels can enhance visibility and encourage role models in non-player roles to support female participation [8]. This is supported by other international sport management research which discusses the role of sport in shaping cultural discourse and processes that drive and facilitate change [39]. However, the culture of sport still perpetuates sexism including diminishing and objectifying women’s capabilities [40]. Another example is sexist language towards women and girls which often reinforces the position of men dominating the sporting landscape [41]. We need continued sport policy and strategies utilising a top-down and bottom-up approach that supports women and girls in non-playing roles within sport for increased diversity in decision making.

There are some limitation in this study to be acknowledged. Firstly, the survey was limited to persons aged 15 years or more. However, non-playing roles as presented in this study are most likely adults. Further, the survey like all survey-based research is likely to include a response bias. Those people engaged in sport are probably more likely to agree to participate in a survey related to participation in sport [42].

## Conclusions

In conclusion, aligned with strategic policy and investment strategies, representation of women in non-playing sport roles have gradual increased. However, women are still underrepresented in terms of coaches, officials and administrators compared to males, but are more likely to be managers. This study highlights that women are more likely to be involved in non-playing roles if they themselves are active or have young children who participate in sport. It is recommended that there is continued mentoring, identification and emphasising of female role models, and other strategies to increase female presence in non-playing roles. This is important for not only the non-playing roles, but also for women and girls to participate throughout the sport ecosystem. Change can occur, but it takes time. Further, we recommend that future research, in line with appropriate gender and cultural-change theories, investigates and discusses the progress of gender equality throughout playing and non-playing role in sport.

## Data Availability

The data were provided by Sport Australia and access to the data would need to be sought from Sport Australia. The research team have a data agreement with Sport Australia to conduct analysis and reporting of AusPlay data.
